# Supplementary material for: Modeling glioblastoma heterogeneity as a dynamic network of cell states
Source: Mol Syst Biol. 2021 Sep 16;17(9):e10105. doi: 10.15252/msb.202010105 (PMC8444284; doi:10.15252/msb.202010105)
Supplement: Supplementary file 5 — Source Data for Figure 3 [file MSB-17-e10105-s001.zip › Figure3A_sourcedata/GSEA_3065/hallmarks_state2.GseaPreranked.1623416269049/HALLMARK_COAGULATION.html]

Details for gene set HALLMARK\_COAGULATION[GSEA]

|  || Dataset | state2 |
| Phenotype | NoPhenotypeAvailable |
| Upregulated in class | na\_neg |
| GeneSet | HALLMARK\_COAGULATION |
| Enrichment Score (ES) | -0.6297888 |
| Normalized Enrichment Score (NES) | -2.1779895 |
| Nominal p-value | 0.0 |
| FDR q-value | 0.0 |
| FWER p-Value | 0.0 |
Table: GSEA Results Summary

  

Fig 1: Enrichment plot: HALLMARK\_COAGULATION      
 Profile of the Running ES Score & Positions of GeneSet Members on the Rank Ordered List

  

| PROBE | GENE SYMBOL | GENE\_TITLE | RANK IN GENE LIST | RANK METRIC SCORE | RUNNING ES | CORE ENRICHMENT || 1 | RAC1 |  |  | 166 | 0.301 | 0.0270 | No |
| 2 | GNB2 |  |  | 432 | 0.193 | 0.0275 | No |
| 3 | GNG12 |  |  | 1154 | 0.108 | -0.0334 | No |
| 4 | DUSP6 |  |  | 1345 | 0.095 | -0.0395 | No |
| 5 | PEF1 |  |  | 1718 | 0.076 | -0.0678 | No |
| 6 | WDR1 |  |  | 1915 | 0.068 | -0.0786 | No |
| 7 | RABIF |  |  | 2395 | 0.051 | -0.1221 | No |
| 8 | CRIP2 |  |  | 3028 | 0.033 | -0.1845 | No |
| 9 | USP11 |  |  | 3142 | 0.031 | -0.1919 | No |
| 10 | TFPI2 |  |  | 3215 | 0.029 | -0.1953 | No |
| 11 | MSRB2 |  |  | 3605 | 0.022 | -0.2334 | No |
| 12 | ACOX2 |  |  | 3627 | 0.022 | -0.2324 | No |
| 13 | ARF4 |  |  | 3841 | 0.018 | -0.2524 | No |
| 14 | DUSP14 |  |  | 4320 | 0.010 | -0.3019 | No |
| 15 | LTA4H |  |  | 4370 | 0.009 | -0.3057 | No |
| 16 | SIRT2 |  |  | 4480 | 0.008 | -0.3162 | No |
| 17 | CAPN5 |  |  | 4827 | 0.002 | -0.3527 | No |
| 18 | ISCU |  |  | 4841 | 0.002 | -0.3538 | No |
| 19 | CSRP1 |  |  | 6030 | -0.017 | -0.4778 | No |
| 20 | FYN |  |  | 6106 | -0.018 | -0.4831 | No |
| 21 | MMP11 |  |  | 6202 | -0.020 | -0.4903 | No |
| 22 | SH2B2 |  |  | 6391 | -0.023 | -0.5068 | No |
| 23 | LGMN |  |  | 6457 | -0.025 | -0.5101 | No |
| 24 | FURIN |  |  | 6544 | -0.026 | -0.5154 | No |
| 25 | CTSO |  |  | 7105 | -0.039 | -0.5692 | No |
| 26 | ANXA1 |  |  | 7265 | -0.044 | -0.5796 | No |
| 27 | PREP |  |  | 7295 | -0.045 | -0.5761 | No |
| 28 | TIMP3 |  |  | 7309 | -0.045 | -0.5708 | No |
| 29 | CPQ |  |  | 7468 | -0.050 | -0.5803 | No |
| 30 | F3 |  |  | 7662 | -0.055 | -0.5927 | No |
| 31 | A2M |  |  | 7912 | -0.063 | -0.6098 | No |
| 32 | BMP1 |  |  | 8090 | -0.071 | -0.6181 | Yes |
| 33 | FBN1 |  |  | 8107 | -0.072 | -0.6092 | Yes |
| 34 | PLAU |  |  | 8131 | -0.073 | -0.6007 | Yes |
| 35 | CAPN2 |  |  | 8165 | -0.075 | -0.5930 | Yes |
| 36 | MMP15 |  |  | 8489 | -0.095 | -0.6134 | Yes |
| 37 | C1R |  |  | 8644 | -0.107 | -0.6140 | Yes |
| 38 | PROS1 |  |  | 8687 | -0.110 | -0.6021 | Yes |
| 39 | THBS1 |  |  | 8789 | -0.120 | -0.5950 | Yes |
| 40 | S100A13 |  |  | 8880 | -0.133 | -0.5848 | Yes |
| 41 | PRSS23 |  |  | 8975 | -0.145 | -0.5732 | Yes |
| 42 | PLAT |  |  | 9145 | -0.176 | -0.5650 | Yes |
| 43 | HTRA1 |  |  | 9178 | -0.184 | -0.5410 | Yes |
| 44 | GSN |  |  | 9238 | -0.206 | -0.5168 | Yes |
| 45 | CTSB |  |  | 9255 | -0.215 | -0.4865 | Yes |
| 46 | ADAM9 |  |  | 9285 | -0.226 | -0.4560 | Yes |
| 47 | MMP2 |  |  | 9307 | -0.235 | -0.4233 | Yes |
| 48 | CD9 |  |  | 9324 | -0.248 | -0.3881 | Yes |
| 49 | LRP1 |  |  | 9338 | -0.259 | -0.3510 | Yes |
| 50 | FN1 |  |  | 9364 | -0.294 | -0.3099 | Yes |
| 51 | LAMP2 |  |  | 9380 | -0.322 | -0.2636 | Yes |
| 52 | SPARC |  |  | 9384 | -0.332 | -0.2146 | Yes |
| 53 | TIMP1 |  |  | 9387 | -0.334 | -0.1651 | Yes |
| 54 | CLU |  |  | 9430 | -0.547 | -0.0883 | Yes |
| 55 | CTSV |  |  | 9434 | -0.601 | 0.0006 | Yes |
Table: GSEA details [plain text format]

  

Fig 2: HALLMARK\_COAGULATION: Random ES distribution      
 Gene set null distribution of ES for **HALLMARK\_COAGULATION**
